# Preoperative Psychological Factors and Early Postoperative Pain After Posterior Spinal Fusion for Scoliosis: A Retrospective Preliminary Study

**DOI:** 10.3390/medicina62040698

**Published:** 2026-04-05

**Authors:** Sergio De Salvatore, Gianmichele Di Cosimo, Michele Inverso, Paolo Brigato, Leonardo Oggiano, Sergio Sessa, Davide Palombi, Francesca Palmieri, Stefano Guida, Antonio Contursi, Caterina Fumo, Pier Francesco Costici

**Affiliations:** 1 Orthopedic and Traumatology Unit, Department of Surgery, Bambino Gesù Children’s Hospital, 00050 Rome, Italy; 2 Department of Anesthesia and Critical Care, IRCCS, Bambino Gesù Children’s Hospital, 00165 Rome, Italy; 3 Research Unit of Orthopaedic and Trauma Surgery, Department of Medicine and Surgery, Università Campus Bio-Medico di Roma, Via Alvaro del Portillo, 21, 00128 Rome, Italy; paolo.brigato@outlook.it (P.B.);

**Keywords:** adolescent idiopathic scoliosis, posterior spinal fusion, postoperative pain, pediatric spine surgery, preoperative anxiety, pain catastrophizing, movement-evoked pain, linear mixed-effects model, risk stratification

## Abstract

*Background and Objectives*: Postoperative pain after posterior spinal fusion (PSF) for adolescent idiopathic scoliosis (AIS) shows substantial interindividual variability, particularly during early mobilization. Although preoperative psychological vulnerability has been associated with less favorable pain trajectories in prior AIS research, evidence focused on the acute postoperative phase remains limited. This preliminary study evaluated whether preoperative psychological factors are associated with acute postoperative pain intensity, with separate assessment of resting and standing pain. *Materials and Methods*: A single-center retrospective cohort study included consecutive adolescents with AIS (<18 years) who underwent primary elective posterior instrumented spinal fusion between 1 January 2024 and 31 December 2025. Preoperative psychological variables were collected using validated instruments (STAIC-State, STAIC-Trait, Pain Catastrophizing Scale, HAQ/FDI, and inverted SRS-22). Pain intensity (VAS 0–10) was recorded at postoperative day (POD) 1, POD2, POD3, discharge, and 2-week follow-up in supine and standing positions. Derived endpoints included peak in-hospital standing pain, in-hospital standing pain burden (AUC), and standing–rest pain gaps. The prespecified inferential analysis used a linear mixed-effects model with fixed effects for time, position, preoperative STAIC-State, and position × STAIC-State interaction, with a patient-level random intercept. *Results*: Thirty-five patients were analyzed (mean age 15.2 ± 3.4 years; 62.9% female), with complete pain data at all timepoints. During hospitalization, standing pain was descriptively higher than resting pain (largest mean difference at POD2: 0.73 VAS points), with convergence at week 2 (both 1.52). In mixed-model analysis, pain significantly decreased at week 2 versus POD1 (β = −1.261, 95% CI −1.853 to −0.669; *p* < 0.001). Preoperative STAIC-State was not independently associated with postoperative pain (β = 0.030, 95% CI −0.065 to 0.124; *p* = 0.545), and no significant position × STAIC-State interaction was found (β = −0.008, 95% CI −0.079 to 0.064; *p* = 0.836). *Conclusions*: In this retrospective preliminary AIS cohort, postoperative pain improved significantly over time, while movement-evoked pain remained relevant during early recovery. In this preliminary cohort, no clear association was detected between preoperative state anxiety and acute postoperative pain intensity, supporting the need for broader multidimensional prognostic models in future prospective multicenter studies.

## 1. Introduction

Adolescent idiopathic scoliosis (AIS) is the most common spinal deformity in otherwise healthy adolescents and remains a major indication for posterior spinal fusion (PSF) when curve progression and deformity burden exceed conservative thresholds [1]. Although modern instrumentation and perioperative protocols have improved surgical safety, early postoperative recovery after PSF is still characterized by substantial interindividual variability in pain intensity, functional pain during mobilization, and early recovery trajectories. In adolescent populations, this variability is clinically meaningful because high early pain may delay mobilization, impair participation in rehabilitation, increase analgesic requirements, and potentially influence the transition from acute to persistent postsurgical pain [2,3].

AIS has also been associated with altered body image, self-perception, and psychosocial burden [4]. In addition, some pathophysiological models have proposed abnormalities in sensorimotor integration and body schema as possible contributors to AIS. Although these hypotheses remain evolving, they provide a plausible conceptual framework linking deformity, psychoemotional characteristics, and pain perception [5,6]. The available AIS literature suggests that postoperative pain is not uniformly distributed across patients. In multicenter and longitudinal cohorts, preoperative pain burden has been associated with postoperative pain outcomes, and non-negligible proportions of patients report clinically relevant pain beyond the immediate postoperative period [2]. Notably, trajectory-based analyses have identified distinct postoperative pain phenotypes, with presurgical characteristics, including psychosocial dimensions, contributing to between-patient heterogeneity. These observations support the concept that postoperative pain after PSF is not only a surgical phenomenon, but also a multidimensional response shaped by baseline patient-level factors [3].

Within this framework, preoperative psychological vulnerability has emerged as a biologically and clinically plausible determinant of pain amplification and pain persistence. Prospective AIS-focused research has shown that child anxiety is associated with less favorable pain trajectories and with persistent postsurgical pain risk, alongside perioperative nociceptive burden and procedural factors [3]. Subsequent studies analyzing acute postoperative trajectories after spinal fusion further reinforced the relevance of individual preoperative profiles for early pain phenotypes and downstream outcomes [5]. In parallel, work on pain catastrophizing in AIS has shown that catastrophic pain cognitions are associated with poorer patient-reported outcomes in the perioperative setting, suggesting that cognitive-affective factors may meaningfully modulate recovery quality [7,8].

Despite increasing recognition of these associations, important gaps remain in the acute phase literature. Many studies emphasize medium- or long-term endpoints, while fewer analyses focus on the first postoperative days, when analgesic decisions and mobilization tolerance are most actionable. Moreover, acute pain is frequently treated as a single construct, whereas resting pain and movement-evoked pain may reflect partially different mechanisms and may not be equally sensitive to psychological predictors. Lastly, most available datasets are heterogeneous in perioperative management, which complicates interpretation of predictor–outcome relationships. Therefore, there is a need for focused analyses in clinically homogeneous cohorts with standardized perioperative care and repeated early pain measurements to clarify whether preoperative psychological factors are associated with acute postoperative pain expression.

The present preliminary report addresses this question by evaluating the association between preoperative psychological factors (state/trait anxiety, pain catastrophizing, functional impairment, and scoliosis-related health status) and acute postoperative pain after PSF for scoliosis. Pain was assessed longitudinally during hospitalization and early follow-up, with separate consideration of resting and standing pain to capture both baseline nociception and mobilization-related pain burden. The working hypothesis was that higher preoperative psychological vulnerability would be associated with higher acute postoperative pain intensity, particularly for standing pain during early mobilization.

## 2. Methods

### 2.1. Study Design and Setting

This study was designed as a single-center retrospective cohort analysis and is reported in accordance with the STROBE recommendations for observational studies. The study was conducted at a tertiary pediatric spine center and included patients who underwent posterior spinal fusion for AIS during the predefined study period. The upper age limit of 18 years was selected to reflect both the clinical definition of AIS and the institutional care pathway for adolescent spinal deformity surgery. Although AIS typically develops earlier in adolescence, patients up to 18 years of age are routinely managed within this clinical category at our center. Because of the limited sample size, the cohort was analyzed as a whole and no sex-stratified inferential analysis was performed.

### 2.2. Study Period and Patient Selection

All consecutive eligible patients who underwent surgery between 1 January 2024 and 31 December 2025 were identified through institutional electronic records. Patients were included if they had:

• Diagnosis of AIS;
• Younger than 18 years at the time of surgery;
• Underwent primary elective posterior instrumented spinal fusion at the study center;
• Complete postoperative data for the prespecified outcomes and assessment timepoints.

Patients were excluded if they:

• Were 18 years of age or older;
• Were non-idiopathic scoliosis (including congenital, neuromuscular, syndromic, or other secondary deformities);
• Had undergone revision surgery or previous major spinal procedures;
• Were managed with a non-comparable perioperative pathway;
• Had missing or incomplete data for the primary postoperative endpoints.

Due to the retrospective design, exclusion for psychiatric comorbidities or chronic pain syndromes was limited to conditions clearly documented in the clinical record; no systematic stratification for these variables was possible in the present dataset. Moreover, only patients with complete postoperative pain assessments at all prespecified timepoints were retained for the present analysis. This criterion was adopted to ensure a consistent longitudinal dataset for repeated-measures modelling. However, it may have introduced selection bias by excluding otherwise eligible patients with incomplete follow-up documentation.

### 2.3. Data Collection and Baseline Variables

Clinical and questionnaire data were retrospectively extracted from institutional electronic records using a standardized abstraction procedure and anonymized before analysis. Baseline variables included age, sex, preoperative major Cobb angle, and curve pattern according to the original Lenke classification [9]. Operative time and estimated blood loss were also collected as perioperative descriptors. Preoperative psychological variables were collected from routinely administered validated instruments (Section 2.4).

### 2.4. Preoperative Psychological Variables

Preoperative psychological status was assessed using routinely collected, validated patient-reported instruments selected to capture complementary domains potentially relevant to postoperative pain. The State–Trait Anxiety Inventory for Children (STAIC-State and STAIC-Trait) was used to distinguish situational preoperative anxiety from a more stable dispositional anxiety profile. The Pain Catastrophizing Scale (PCS) was used to assess maladaptive pain-related cognitions. Functional limitation was assessed using the HAQ/FDI score, and scoliosis-specific health status was assessed using the SRS-22 questionnaire. To maintain directional consistency across predictors, the SRS-22 score was inverted (132 minus the raw score), so that higher values indicated worse baseline status across all psychological and health-status measures considered in the analysis.

### 2.5. Perioperative Analgesia and Antiemetic Protocol

During the study period, perioperative pain management followed a standardized institutional protocol for AIS patients undergoing posterior spinal fusion. Intraoperatively and during the immediate postoperative ICU phase (POD0), analgesia was based on an elastomeric infusion pump delivering tramadol, ketorolac, and ondansetron, together with adjunctive normal saline. Additional perioperative medications included dexamethasone, magnesium, remifentanil during anesthesia, scheduled paracetamol, omeprazole, and, when indicated, morphine bolus administration. Bilateral erector spinae plane block was performed with ropivacaine 0.1% plus dexmedetomidine.

During POD1, the elastomeric infusion pump was continued with tramadol, ketorolac, and ondansetron, and patients also received dexamethasone, dimenhydrinate, scheduled paracetamol, omeprazole, and cefazolin according to institutional practice. On POD2, the elastomeric regimen was de-escalated to ketorolac alone, while supportive medications were continued; drain and urinary catheter removal and post-mobilization radiography were also performed according to routine care. From POD3 onward, analgesic therapy was progressively tapered until discharge.

Although perioperative management followed a shared institutional pathway, some treatment-level variability remained, including clinically indicated individualized adjustments and the use of regional analgesia in selected patients. Accordingly, the cohort should be considered clinically homogeneous at the pathway level rather than strictly identical in all analgesic exposures.

### 2.6. Outcomes

The primary outcome was postoperative pain intensity measured on a 0–10 visual analog scale (VAS). Pain was recorded at POD1, POD2, POD3, discharge, and 2-week follow-up in both supine and standing conditions to distinguish resting nociception from movement-evoked pain. Pain assessed on POD1, POD2, and POD3 was considered part of the acute postoperative phase, whereas pain assessed at the 2-week follow-up was interpreted as an early subacute recovery outcome. Derived endpoints included peak in-hospital standing pain, in-hospital standing pain burden (AUC), peak standing–rest gap during hospitalization and standing–rest gap at discharge and at 2 weeks.

### 2.7. Statistical Analysis

Continuous variables are presented as mean ± standard deviation (SD), and categorical variables as counts and percentages, as appropriate. The primary inferential analysis was prespecified to evaluate the association between preoperative STAIC-State and postoperative pain trajectories. Other collected variables, including clinical baseline characteristics (age, sex, main Cobb angle, operative time, and estimated blood loss) and additional psychological measures (STAIC-Trait, PCS, HAQ/FDI, and inverted SRS-22), were not entered simultaneously into the primary mixed-effects model because of the limited sample size and the resulting risk of overfitting. These additional psychological variables were instead examined in exploratory univariable analyses against selected postoperative pain endpoints.

No a priori sample size calculation was performed because this was a retrospective preliminary study based on all consecutive eligible patients treated during the predefined study period. Accordingly, the inferential analyses should be regarded as exploratory and hypothesis-generating rather than definitive.

The primary model consisted of a linear mixed-effects analysis with postoperative VAS pain score as the dependent variable and fixed effects for time, position (supine vs. standing), preoperative STAIC-State, and the interaction between position and preoperative STAIC-State. A patient-level random intercept was included to account for within-subject correlation across repeated measurements. Given the modest sample size, the model was intentionally parsimonious and was not designed to provide a fully adjusted estimate of the independent association between psychological variables and postoperative pain.

Additional psychological variables were examined in exploratory univariable analyses against selected postoperative pain endpoints. These analyses were performed to characterize potential signal direction and magnitude, but were not intended to support formal causal inference. Results are reported as beta coefficients with 95% confidence intervals and two-sided *p*-values. Statistical significance was set at *p* < 0.05. No imputation was performed. All analyses were conducted in R (R Foundation for Statistical Computing, Vienna, Austria).

### 2.8. Ethics

This study was conducted in accordance with the Declaration of Helsinki and applicable national and international data protection regulations. Ethical review and approval were not required for this study, in accordance with Regulation (EU) 2016/679 (GDPR), Italian Legislative Decree 101/2018, and the guidance of the Italian Data Protection Authority, because the study was retrospective and based exclusively on fully anonymized data collected during routine clinical practice. For the same reason, patient consent was waived.

## 3. Results

### 3.1. Study Population and Baseline Characteristics

A total of 35 adolescents with AIS were included in the analysis. The cohort was predominantly female (22/35, 62.9%), with a mean age of 15.2 ± 3.4 years. Mean preoperative main Cobb angle was 63.3 ± 20.0°, indicating overall moderate-to-severe deformity. Curve patterns were heterogeneous, with Lenke type 1 being the most frequent (42.9%), followed by types 3 (25.7%), 6 (11.4%), 2 (8.6%), 5 (8.6%), and 4 (2.9%). Baseline patient characteristics and preoperative psychological measures are summarized in Table 1. All included patients had complete pain assessments for both positions at all prespecified postoperative timepoints and were therefore eligible for longitudinal analysis without imputation.

Regarding baseline psychological profile, mean scores were 54.00 ± 5.35 for STAIC-State, 58.67 ± 7.89 for STAIC-Trait, 23.60 ± 5.42 for PCS, and 34.07 ± 9.33 for HAQ/FDI. Mean inverted SRS-22 score was 71.40 ± 9.95.

### 3.2. Postoperative Pain Trajectory by Position

Postoperative VAS values by timepoint and position are reported in Table 2. Across in-hospital assessments, standing pain was descriptively higher than resting pain. Mean resting VAS was 2.36 ± 1.53 at POD1 and POD2, 2.59 ± 1.62 at POD3, and 2.27 ± 1.20 at discharge. Mean standing VAS was 2.91 ± 1.19 at POD1, 3.09 ± 1.51 at POD2, 2.86 ± 1.52 at POD3, and 2.73 ± 1.45 at discharge.

**Figure 1:**
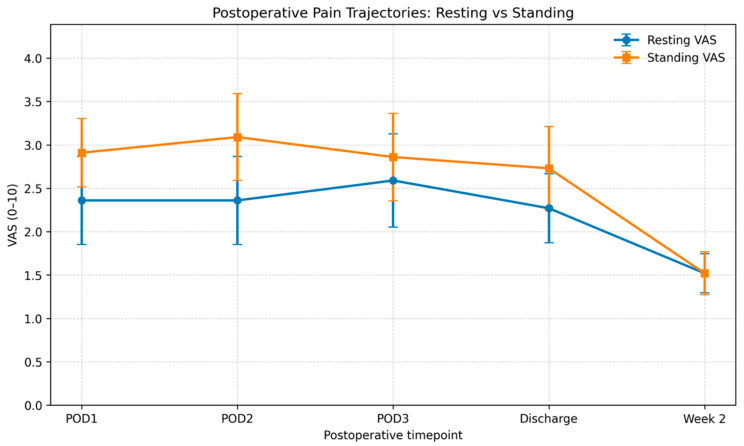
Postoperative pain trajectories by position.

**Figure 2:**
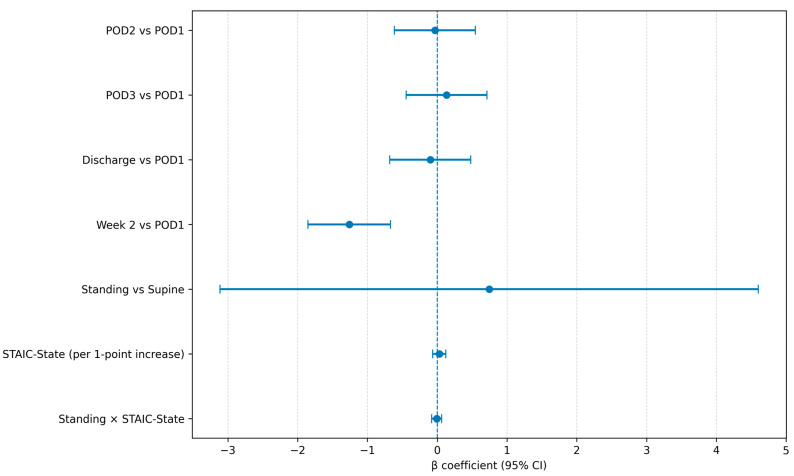
Mixed-effects model estimates for postoperative VAS pain.

The standing–rest difference was greatest at POD2 (0.73) and progressively narrowed thereafter (0.27 at POD3 and 0.46 at discharge). At Week 2, pain decreased in both positions and converged (resting VAS 1.52 ± 0.68; standing VAS 1.52 ± 0.75; standing–rest difference 0.00). Pain trajectories are reported in Figure 1 and Figure 2.

### 3.3. Derived Pain Endpoints

Derived postoperative pain endpoints are shown in Table 3. Mean peak standing pain during hospitalization (POD1–discharge) was 4.05 ± 1.29. Mean in-hospital standing pain burden (AUC) was 8.77 ± 3.19. The peak standing–rest gap during hospitalization was 1.59 ± 0.96. This positional gap decreased at discharge (0.45 ± 1.30) and was negligible at Week 2 (0.00 ± 0.89).

### 3.4. Association Between Preoperative State Anxiety and Postoperative Pain

Results of the linear mixed-effects model are reported in Table 4. Compared with POD1, pain at Week 2 was significantly lower (β = −1.261, 95% CI −1.853 to −0.669; *p* < 0.001), indicating a significant reduction in postoperative pain over time. No significant differences were observed for POD2, POD3, or discharge versus POD1.

Preoperative STAIC-State score was not significantly associated with postoperative VAS (β = 0.030, 95% CI −0.065 to 0.124; *p* = 0.545). The position effect (standing vs. supine) was not statistically significant (β = 0.744, 95% CI −3.113 to 4.601; *p* = 0.706), and the position × STAIC-State interaction was also non-significant (β = −0.008, 95% CI −0.079 to 0.064; *p* = 0.836). Overall, the model identified a significant reduction in pain over time, whereas no clear association between preoperative state anxiety and postoperative pain intensity was detected within the constraints of this preliminary parsimonious model.

In additional exploratory univariable analyses, the other preoperative psychological variables collected in the study (STAIC-Trait, PCS, HAQ/FDI, and inverted SRS-22) were tested against selected postoperative pain endpoints, including peak standing pain during hospitalization, in-hospital standing pain burden (AUC), and standing pain at discharge. No statistically significant associations emerged for any of these measures in this preliminary sample (Table 5). These findings should be interpreted cautiously given the limited sample size and the exploratory nature of the analyses.

## 4. Discussion

The present preliminary study evaluated whether preoperative psychological vulnerability, primarily captured by state anxiety, was associated with early postoperative pain after posterior spinal fusion for AIS. Firstly, postoperative pain decreased significantly over time, with the clearest improvement observed at the 2-week follow-up compared with POD1. Moreover, during hospitalization, standing pain was descriptively higher than resting pain, supporting the clinical relevance of movement-evoked pain as a distinct component of early recovery. Lastly, preoperative STAIC-State was not independently associated with postoperative pain intensity in the mixed-effects model, and no significant interaction was observed between state anxiety and pain position. Exploratory analyses likewise did not identify significant associations for PCS, STAIC-Trait, HAQ/FDI, or inverted SRS-22. These findings suggest that, in this cohort, a measurable signal linking baseline preoperative psychological vulnerability to early postoperative pain was not detected, although the present dataset does not exclude smaller or more complex associations.

These findings should be interpreted within the broader AIS literature on psychological predictors of pain, which is more heterogeneous than it may initially appear. Not all previous studies have shown that a single preoperative anxiety measure predicts early postoperative pain. In a preliminary study of adolescents undergoing spinal fusion, Ferland et al. found that preoperative trait anxiety was associated with preoperative pain intensity, but not with postoperative pain intensity in the acute or intermediate postoperative period, a pattern that is directionally consistent with the present results [10]. By contrast, other AIS studies have reported associations between psychological factors and less favorable postoperative pain outcomes. However, these investigations have generally examined broader constructs or later recovery phenotypes. Connelly et al. showed that greater baseline anxiety was associated with slower improvement in postoperative pain trajectories over the first 6 months after surgery. Chidambaran et al. found that child anxiety predicted persistent postsurgical pain rather than immediate postoperative pain scores. Similarly, Charalampidis et al. reported that low preoperative mental health predicted persistent postoperative back pain, again pointing toward a longer-term recovery phenotype rather than acute in-hospital pain expression [3,11,12].

A similar distinction applies to the catastrophizing pain. Chabot et al. showed that pain catastrophizing in AIS may vary across the perioperative period, suggesting that it does not necessarily behave as a fixed baseline trait. Ramo et al. found that higher preoperative catastrophizing was associated with worse patient-reported outcomes, including pain-related SRS-30 scores. However, their analysis focused on self-reported health status at 2-year follow-up rather than acute postoperative pain intensity during hospitalization [8,13]. Older work by Lamontagne et al. also suggested a relationship between anxiety and postoperative pain after scoliosis surgery, but that study emphasized postoperative anxiety levels, which may be more proximal to pain experience than a single preoperative state-anxiety assessment [3,11,13,14]. Overall, the apparent discrepancy between the present findings and prior literature therefore seems more likely to reflect differences in psychological construct, timing of assessment, and outcome definition than a true biological contradiction.

Several factors may explain why no independent anxiety signal was detected in the present analysis. Acute postoperative pain after spinal fusion is strongly influenced by perioperative nociceptive load, analgesic exposure, inflammatory response, and early mobilization demands. In addition, although perioperative care followed a common institutional pathway, some variability in analgesic exposure remained, including individualized adjustments and the use of regional analgesia in selected patients. This variability may have reduced or obscured smaller associations between baseline psychological measures and postoperative pain expression. In a clinically homogeneous cohort managed under a standardized perioperative pathway, these factors may attenuate detectable between-patient effects of baseline psychological characteristics. In addition, state anxiety represents only one dimension of psychological vulnerability. Catastrophizing, coping style, sleep quality, preoperative pain history, and family-related psychosocial context may interact in a multidimensional fashion and are unlikely to be fully captured by a single baseline anxiety measure [15,16]. The present findings therefore add nuance rather than contradiction to the literature. Psychological factors may still matter, but their influence on pain may become more evident when assessed through broader constructs, dynamic perioperative measures, or longer-term recovery trajectories [2,5,7,8,,^17^].

The distinction between resting and standing pain deserves specific emphasis. During hospitalization, standing pain was descriptively higher than resting pain, with gradual convergence over time and no residual difference at 2 weeks. This pattern should be interpreted as descriptive rather than as evidence of a robust independent position effect within the mixed model. Nevertheless, it supports the clinical view that movement-evoked pain captures a more functional dimension of recovery than static pain at rest. Postoperative assessment protocols in AIS should continue to report both resting and mobilization-related pain, since rehabilitation tolerance and discharge readiness depend more on functional pain burden than on supine comfort alone. Even in the absence of significant psychological predictors, the present data reinforce the clinical value of this dual pain assessment approach.

From a translational perspective, the present results do not support reliance on preoperative STAIC-State as a stand-alone tool for acute postoperative pain stratification in this clinical setting. This should not, however, be interpreted as evidence against psychosocial assessment in AIS surgery. The broader literature continues to indicate that psychosocial burden is relevant to recovery quality and longer-term outcomes [12,18]. A more clinically useful approach may be to integrate psychological screening into multivariable perioperative risk models that also account for deformity severity, preoperative pain burden, analgesic exposure, and early functional recovery. These models may better capture the multifactorial nature of postoperative pain than isolated single-domain predictors.

Finally, a broader interpretive framework may also be relevant. Beyond its structural deformity, AIS has been associated with altered body image, psychosocial distress, and reduced self-perception of trunk alignment [6]. Some authors have also proposed that abnormalities in sensorimotor integration and body schema may contribute to the clinical expression of the deformity [19,20]. Although these models remain exploratory and should not be overstated, they support the idea that pain perception in AIS may be shaped not only by surgical and nociceptive factors, but also by body representation and psychoemotional context [21]. Within this perspective, the present findings suggest that a single preoperative anxiety measure may simply be too narrow to capture the multidimensional mechanisms influencing early postoperative pain expression [6,19].

The study also offers methodological strengths that improve interpretability. The cohort was clinically homogeneous, focusing on patients treated with posterior fusion for AIS, and postoperative pain was assessed at predefined timepoints in both supine and standing conditions. Complete pain data across all scheduled assessments reduced uncertainty related to missing-outcome mechanisms. The use of a linear mixed-effects framework appropriately addressed within-subject correlation and allowed simultaneous estimation of temporal change, positional effect, and anxiety-related terms within a single inferential structure.

### Limitations

Several limitations should be considered when interpreting these findings. The retrospective single-center design limits causal inference and may reduce external validity across institutions with different perioperative practices. The sample size was modest, and the study was explicitly preliminary and no a priori sample size calculation was performed. Moreover, the primary inferential model was intentionally parsimonious and was not designed to provide a fully adjusted estimate incorporating all potentially relevant perioperative, surgical, and psychosocial covariates. In addition, although patients were managed within a shared institutional perioperative pathway, treatment-level variability remained, including clinically indicated individualized analgesic adjustments and the use of regional analgesia in selected cases. Only patients with complete postoperative pain assessments at all prespecified timepoints were retained for analysis, which improved longitudinal consistency but may have introduced selection bias. Follow-up was limited to the early postoperative period, preventing conclusions regarding persistent pain trajectories beyond two weeks. In addition, psychiatric comorbidities and chronic pain syndromes could only be identified when clearly documented in the clinical record. Finally, the age range of the cohort may have introduced heterogeneity in psychological maturity and pain appraisal. Accordingly, the present findings should be interpreted as preliminary and hypothesis-generating.

## 5. Conclusions

In this retrospective preliminary cohort of adolescents undergoing posterior spinal fusion for AIS, postoperative pain decreased over the early recovery period, with the clearest improvement observed by the 2-week follow-up. Standing pain was descriptively higher than resting pain during hospitalization, supporting the clinical relevance of movement-evoked pain assessment in routine postoperative monitoring. Within the constraints of a small preliminary sample and a parsimonious mixed-effects model, no clear association was detected between preoperative state anxiety and acute postoperative pain intensity, and no differential effect emerged according to pain position. These findings suggest that early pain expression after AIS fusion is likely shaped by multiple interacting perioperative and patient-level factors rather than by baseline anxiety alone. Larger prospective multicenter studies using broader psychosocial characterization and more fully adjusted perioperative models are warranted to refine individualized pain-risk stratification.

## Figures and Tables

**Table 1:** Baseline patient characteristics and preoperative psychological measures.

Variable	Value
Demographic and preoperative clinical characteristics	
Age (years), mean ± SD	15.2 ± 3.4
Sex, female, n (%)	22 (62.9)
Sex, male, n (%)	13 (37.1)
Main Cobb angle (°), mean ± SD	63.3 ± 20.0
Lenke type 1, n (%)	15 (42.9)
Lenke type 2, n (%)	3 (8.6)
Lenke type 3, n (%)	9 (25.7)
Lenke type 4, n (%)	1 (2.9)
Lenke type 5, n (%)	3 (8.6)
Lenke type 6, n (%)	4 (11.4)
Perioperative variables	
Operative time (min), mean ± SD	227.4 ± 52.9
Estimated blood loss (mL), mean ± SD	447.0 ± 254.4
Preoperative psychological measures	
STAIC-State, mean ± SD	54.00 ± 5.35
STAIC-Trait, mean ± SD	58.67 ± 7.89
Pain Catastrophizing Scale (PCS), mean ± SD	23.60 ± 5.42
HAQ/FDI score, mean ± SD	34.07 ± 9.33
SRS-22 (inverted *), mean ± SD	71.40 ± 9.95

PCS, Pain Catastrophizing Scale; STAIC, State–Trait Anxiety Inventory for Children; HAQ/FDI, functional disability score; SRS-22, Scoliosis Research Society-22 questionnaire. * SRS-22 was transformed as 132—raw score; higher values indicate worse baseline status.

**Table 2:** VAS by timepoint and position.

Timepoint	Resting VAS (Mean ± SD)	Standing VAS (Mean ± SD)	Standing—Rest (Descriptive)
POD1	2.36 ± 1.53	2.91 ± 1.19	0.55
POD2	2.36 ± 1.53	3.09 ± 1.51	0.73
POD3	2.59 ± 1.62	2.86 ± 1.52	0.27
Discharge	2.27 ± 1.20	2.73 ± 1.45	0.46
Week 2	1.52 ± 0.68	1.52 ± 0.75	0.00

POD: Postoperative Day.

**Table 3:** Derived VAS endpoints.

Derived Endpoint	Mean ± SD
Peak standing VAS in hospital (POD1–discharge)	4.05 ± 1.29
In-hospital standing pain burden (AUC, POD1–discharge)	8.77 ± 3.19
Peak standing–rest gap in hospital	1.59 ± 0.96
Standing–rest gap at discharge	0.45 ± 1.30
Standing–rest gap at Week 2	0.00 ± 0.89

POD: Postoperative Day

**Table 4:** Linear mixed-effects model for postoperative VAS pain.

Fixed Effect	β (95% CI)	*p*-Value
Intercept	0.897 (−4.232 to 6.026)	0.735
POD2 vs. POD1	−0.033 (−0.613 to 0.546)	0.910
POD3 vs. POD1	0.133 (−0.446 to 0.713)	0.653
Discharge vs. POD1	−0.100 (−0.680 to 0.480)	0.736
Week 2 vs. POD1	**−1.261 (−1.853 to −0.669)**	**<0.001**
Standing vs. Supine	0.744 (−3.113 to 4.601)	0.706
STAIC-State (per 1-point increase)	0.030 (−0.065 to 0.124)	0.545
Standing × STAIC-State	−0.008 (−0.079 to 0.064)	0.836

POD, postoperative day; CI, confidence interval; Bold, its significative.

**Table 5:** Association between preoperative psychological measures and early postoperative pain outcomes.

Preoperative Predictor	Peak Standing VAS (In-Hospital) β (95% CI)	*p*-Value	AUC Standing VAS (In-Hospital) β (95% CI)	*p*-Value	Standing VAS at Discharge β (95% CI)	*p*-Value
STAIC-State	0.067 (−0.085 to 0.220)	0.356	0.060 (−0.332 to 0.452)	0.675	0.001 (−0.097 to 0.099)	0.978
STAIC-Trait	0.040 (−0.064 to 0.144)	0.424	0.077 (−0.186 to 0.340)	0.777	−0.036 (−0.098 to 0.025)	0.224
PCS	0.041 (−0.113 to 0.194)	0.576	0.080 (−0.305 to 0.465)	0.717	0.043 (−0.049 to 0.134)	0.332
HAQ/FDI	0.049 (−0.036 to 0.135)	0.236	0.134 (−0.077 to 0.345)	0.926	−0.014 (−0.069 to 0.041)	0.600
Inverted SRS-22	−0.063 (−0.139 to 0.013)	0.098	−0.150 (−0.342 to 0.041)	0.158	−0.036 (−0.085 to 0.012)	0.128

VAS, visual analog scale

## Data Availability

The original contributions presented in this study are included in the article. Further inquiries can be directed to the corresponding author.
